# The GLP-1 agonist, liraglutide, ameliorates inflammation through the activation of the PKA/CREB pathway in a rat model of knee osteoarthritis

**DOI:** 10.1186/s12950-019-0218-y

**Published:** 2019-06-06

**Authors:** Qihong Que, Xinghua Guo, Longxin Zhan, Shaodong Chen, Zengli Zhang, Xiaoming Ni, Bin Ye, Shuanglin Wan

**Affiliations:** 1 Department of Orthopedic Surgery, Hospital of Traditional Chinese Medicine of Songyang, Zhejiang, 323400 People’s Republic of China; 2 Department of Internal Medicine, Hospital of Traditional Chinese Medicine of Songyang, Zhejiang, 323400 People’s Republic of China; 3 Department of Orthopedic Surgery, Lishui City People’s Hospital, Zhejiang, 323000 People’s Republic of China; 4 Department of Endocrinology, Lishui City People’s Hospital, Zhejiang, 323000 People’s Republic of China; 5 Department of Orthopedic, Sir Run Run Shaw Hospital of Hangzhou, No.3, Qingchun east road, Jianggan district, Hangzhou Zhejiang, 310020 People’s Republic of China

**Keywords:** GLP-1R, Liraglutide, PKA/CREB pathway, Inflammation, Osteoarthritis

## Abstract

**Background:**

Inflammation is a common pathological phenomenon of osteoarthritis (OA). Accumulated evidence indicates that ameliorating or suppressing inflammation might be a promising and effective therapeutic strategy for the treatment of OA. Notably, glucagon-like peptide-1 (GLP-1)-based drugs are being successfully used to control glucose levels in patients with diabetes mellitus. In addition, recent findings have indicated that GLP-1 agonists, such as liraglutide have therapeutic potential in preventing inflammation-related disorders through the regulation of protein kinase A (PKA)/ cyclic adenosine monophosphate (cAMP) response element-binding protein (CREB) signals. Intra-articular injection of monoiodoacetate (MIA) has been widely used to induce OA. Thus, the present study aimed to investigate whether liraglutide has anti-inflammatory effects on MIA-induced OA rats and uncover its underlying molecular mechanisms.

**Methods:**

Intra-articular injection of MIA was used to induce knee OA in a rat model. Subcutaneous injection of liraglutide was used to upregulate the expression of GLP-1 receptor (GLP-1R). Western blot analysis was utilized to measure the expression of GLP-1R, PKA/CREB pathway components and inflammation-related proteins, such as tumor necrosis factor-alpha (TNF-α), interleukin-1 beta (IL-1β), and IL-6. Immunoprecipitation techniques were used to detect the interactions between GLP-1R and the PKA/CREB pathway.

**Results:**

The levels of GLP-1R decreased significantly in the knees of OA rats, accompanied by the downregulation of PKA /CREB signals and upregulation of inflammation-related proteins. We also found that GLP-1R interacted with the PKA/CREB pathway and that liraglutide could activate PKA/CREB signals, thereby inhibiting the expression of inflammation-related proteins.

**Conclusions:**

Together our results suggest that liraglutide exhibits anti-inflammatory activity through the activation of the PKA/CREB pathway in an OA rat model.

## Background

Osteoarthritis (OA) is one of the most prevalent and debilitating joint diseases. It is pathologically characterized by loss of articular cartilage, hypertrophic bone changes, osteophyte formation, subchondral bone remodeling and low-grade synovial inflammation. In addition, it is clinically characterized by joint pain, nighttime pain, functional impairment and limited movement [1, 2]. Approximately 10% of the world’s population suffers from this disease and it has become the first major cause of human disability [3, 4]. OA can affect the hand, spine, hip, wrist and ankle. However, knee OA represents the most widespread form of this disease [5]. Although, there are various therapeutic methods for OA, such as surgery, drug and adjuvant therapy, it is difficult to completely stop the development of OA, which results in continuous pain and even long-term disability [6]. Thus, it is crucial to elucidate the precise underlying molecular mechanisms mediating its development in order to find novel, efficacious and promising therapeutic modalities for OA.

OA has a multifactorial pathophysiology with mechanical, metabolic and inflammatory contributions to its etiology, with numerous risk factors such as age, gender and joint injury [7]. In particular, accumulating evidence suggests that inflammation is a prominent etiology of OA, and that chondrocytes and synovial cells overproduce many of the soluble inflammatory mediators, such as cytokines, chemokines, growth factors, adipokines, prostaglandins, leukotrienes and regulators such as CPB [7, 8]. Given the important role of inflammation in OA, novel anti-inflammatory therapies could offer new opportunities for treating the disease. For example, Hwang et al., indicated that *Zanthoxylum piperitum* ethanol extract could decrease the levels of reactive oxygen species (ROS) and the expression of hypoxanthine phosphoribosyl transferase 1 (HPRT1), thereby suppressing inflammation. This suggests that this extract might be a potential therapeutic agent to modulate OA [5]. Therefore, molecules involved in inflammation are attractive therapeutic targets for the treatment of OA.

GLP-1 is an incretin hormone produced by intestinal enteroendocrine L cells in order to regulate glucose and energy homeostasis via GLP-1R binding and induction [9]. GLP-1 not only regulates blood glucose levels, but is also closely associated with various pathophysiological processes, such as inflammation. Thus, GLP-1R agonists, such as liraglutide, show beneficial effects on inflammation-related diseases. To this end, Chen et al., has suggested that activation of GLP-1R with liraglutide could protect chondrocytes against endoplasmic reticulum (ER) stress, apoptosis and inflammation by decreasing the release of inflammatory mediators and regulating phosphoinositide 3-kinase (PI3K)/AKT signaling, which suggests that GLP-1R is a therapeutic target for the treatment of OA and that liraglutide could be a therapeutic candidate for clinical application [10]. Moreover, GLP-1R activates adenylyl cyclase (AC) to produce cyclic adenosine monophosphate (cAMP), which in turn activates protein kinase A (PKA) that activates cAMP response element-binding protein (CREB), a constitutively expressed nuclear transcription factor that regulates the expression of genes involved in inflammation [11, 12]. However, to the best of our knowledge, to date, no data has been reported on whether GLP-1R exerts an anti-inflammatory effect on OA through inhibition of the PKA/CREB pathway. Moreover, intra-articular injection of monoiodoacetate (MIA) induces loss of articular cartilage with subchondral bone lesions that mimic those of human OA and This model offers a rapid and minimally invasive method to reproduce OA-like lesions in a rodent species [13]. Thus, in the present study, we wanted to test whether GLP-1R agonist, liraglutide could alleviate inflammation by activating the PKA/CREB pathway in a rat model of knee OA induced by MIA, which might provide therapeutic candidate for the clinical management of OA.

## Materials and methods

### Drugs and chemicals

The GLP-1 agonist, liraglutide was purchased from Novo Nordisk (Princeton, NJ USA). MIA, used to induce OA in rats, was purchased from Sigma-Aldrich (St. Louis, MO, USA). Rabbit anti-GLP-1R, PKA, phospho-PKA (Thr197), CREB, phospho-CREB (Ser 133), tumor necrosis factor-alpha (TNF-α), interleukin-1 beta (IL-1β), and IL-6 primary antibodies were purchased from Abcam (Cambridge, UK). Normal rabbit anti-IgG was purchased from Cell Signaling Technology (Cambridge, MA, USA). Rabbit anti-glyceraldehyde 3-phosphate dehydrogenase (GAPDH) and horseradish peroxidase (HRP)-conjugated goat anti-rabbit IgG were obtained from Proteintech (Chicago, IL, USA). The protein A/G agarose beads were purchased from Santa Cruz Biotechnology (Santa Cruz, CA, USA). Immunohistochemical kits were obtained from Boster Biological Technology Co., Ltd. (Pleasanton, CA, USA).

### Animals

A total of 90 adult male Wistar rats, weighing approximately 200 to 250 g, were purchased from the Animal Experiment Center of the Chinese Academy of Medical Sciences, Beijing, China and were maintained in wooden cages at a temperature of 27 °C–30 °C, with a 12 h light-dark photo-periodicity cycle. Rats were given ad libitum access to food and water. The procedures were approved by the Ethics Committee of the Chinese Academy of Medical Sciences, Beijing, China and conformed to international animal care guidelines. All efforts were made to minimize animal discomfort and the number of animals sacrificed.

### Induction of knee OA

Knee OA was induced according to previous protocols [5, 14]. Briefly, 4 mg of MIA were dissolved in 40 μL of sterile saline and this volume of solution was injected intraarticularly through the patellar ligament of the left knee joint of each rat that had been anesthetized using 50 mg/kg sodium pentobarbital by intraperitoneal injection.

### Experimental design

For this study, we performed two experiments for different purposes. In the first experiment, a total of 60 rats were divided equally into control, OA-1, OA-5, OA-10, OA-20 and OA-28 groups. The animals in the control group received an intraarticular injection of saline instead of MIA. The rats in the OA-1, OA-5, OA-10, OA-20 and OA-28 groups received an intraarticular injection of MIA and were sacrificed after 1, 5, 10, 20 and 28 days, respectively after intraarticular injection of MIA. In the second experiment, a total of 30 rats were divided equally into OA, OA + saline and OA + liraglutide groups. All rats in the second experiment received an intraarticular injection of MIA. A previously published research used liraglutide at 50 μg/kg/day subcutaneously (s.c.) to detect apoptosis and the associated inflammatory response of liraglutide in OA model induced by resection of anterior cruciate ligament (ACL) and meniscus [10]. Similarly, the rats in the OA + liraglutide group received 50 μg/kg/day liraglutide dissolved in saline through subcutaneously (sc) administration. The same volume of saline was administered by injection to the OA + saline group. The liraglutide or saline treatment lasted for 28 days. All these rats were sacrificed 28 days after injection of monoiodoacetate under anesthesia using 50 mg/kg sodium pentobarbital by intraperitoneal injection. Left knee joint specimens were obtained and the muscles around the knee joint quickly removed. The femoral condylar cartilage was removed by circular incision along the femoral edge of the articular capsule. Moreover, the femur and tibial fibula were cut off along the bone surface, the joint capsule was cut longitudinally, and the synovial tissue was separated. The cartilage specimens were then stored in liquid nitrogen until further study.

### Immunoprecipitation (IP)

OA cartilage tissues were collected and triturated, and then lysed on ice for 30 min in lysis buffer containing sodium pyrophosphate and protease inhibitors (Sigma-Aldrich). The lysates were centrifuged at 14,000 g for 15 min and the supernatants were collected. Supernatants containing equal amounts of proteins were incubated with 2.5 mg GLP-1R or normal IgG antibodies overnight at 4 °C. The immunoprecipitates were then harvested using protein A/G agarose beads for 4 h at 4 °C. The beads were then washed and subjected to a 5-min boiling step for denaturation. The immunoprecipitates were subsequently analyzed using Western blot techniques.

### Western blot analysis

Total protein of cartilage tissues were extracted using a radio immunoprecipitation assay (RIPA) buffer containing sodium pyrophosphate and protease inhibitors. The protein concentrations were quantified using a bicinchoninic acid (BCA) reagent according to the manufacturer’s instructions. Harvested proteins or immunoprecipitates were separated using 10% sodium dodecyl sulphate-polyacrylamide gel electrophoresis (SDS-PAGE) then transferred to polyvinylidene difluoride (PVDF) membranes. After the membranes were incubated in blocking buffer at room temperature for 2 h, they were washed 3 times in tris-buffered saline and tween 20 (TBST) solution, 5 min each, and then incubated with primary antibodies overnight at 4 °C. The primary antibodies were diluted in blocking buffer at a ratio of 1:1000. Following 3 washes with TBST, 5 min each, the membranes were incubated with an HRP-conjugated goat anti-rabbit IgG (1:3000) antibody for 1 h. The membranes were then washed 3 times with TBST, 5 min each and analyzed by enhanced chemiluminescence (ECL). Quantification of absorbance of the blots was performed using densitometric scanning with a Fusion-FX7 system (Vilber Lourmat, Collégien, France) with mean optical density of the samples normalized to that of GAPDH.

### Immunohistochemical analysis

The levels of GLP-1R were evaluated in each group using immunohistochemical (IHC) staining. Briefly, a portion of harvested cartilage tissues were used to prepare paraffin sections. After being blocked with 3% H_2_O_2_ for 10 min and 5% of bovine serum albumin (BSA) for 30 min, the sections were deparaffinized and rehydrated. Then, sections were incubated with rabbit anti-GLP-1R (1:50) at 4 °C overnight and a secondary goat anti-rabbit antibody at 37 °C was used for 30 min. The sections were washed in a phosphate-buffered saline (PBS) buffer and incubated in a 3, 3-diaminobenzidine (DAB) solution for 3 min. Hematoxylin was used to counterstain the nuclei. Images were captured usinga light microscope and analyzed using Image-Pro Plus 6.0 software (Media Cybernetics; Rockville, MD, USA), and the integral absorbance values were used to measure the levels of GLP-1R. At least three sections from each specimen were used to measure theexpression levels of GLP-1R.

### Statistical analysis

All data are presented as means ± standard deviation (SD) for each group from at least three independent experiments. Statistical comparisons of the data between each group were conducted using one-way analysis of variance (ANOVA) followed by Tukey’s test (GraphPad Prism). *P* values of less than 0.05 were considered statistically significant.

## Results

### Decreased expression of GLP-1R in the cartilage tissues of the knee OA model

**Fig. 1:**
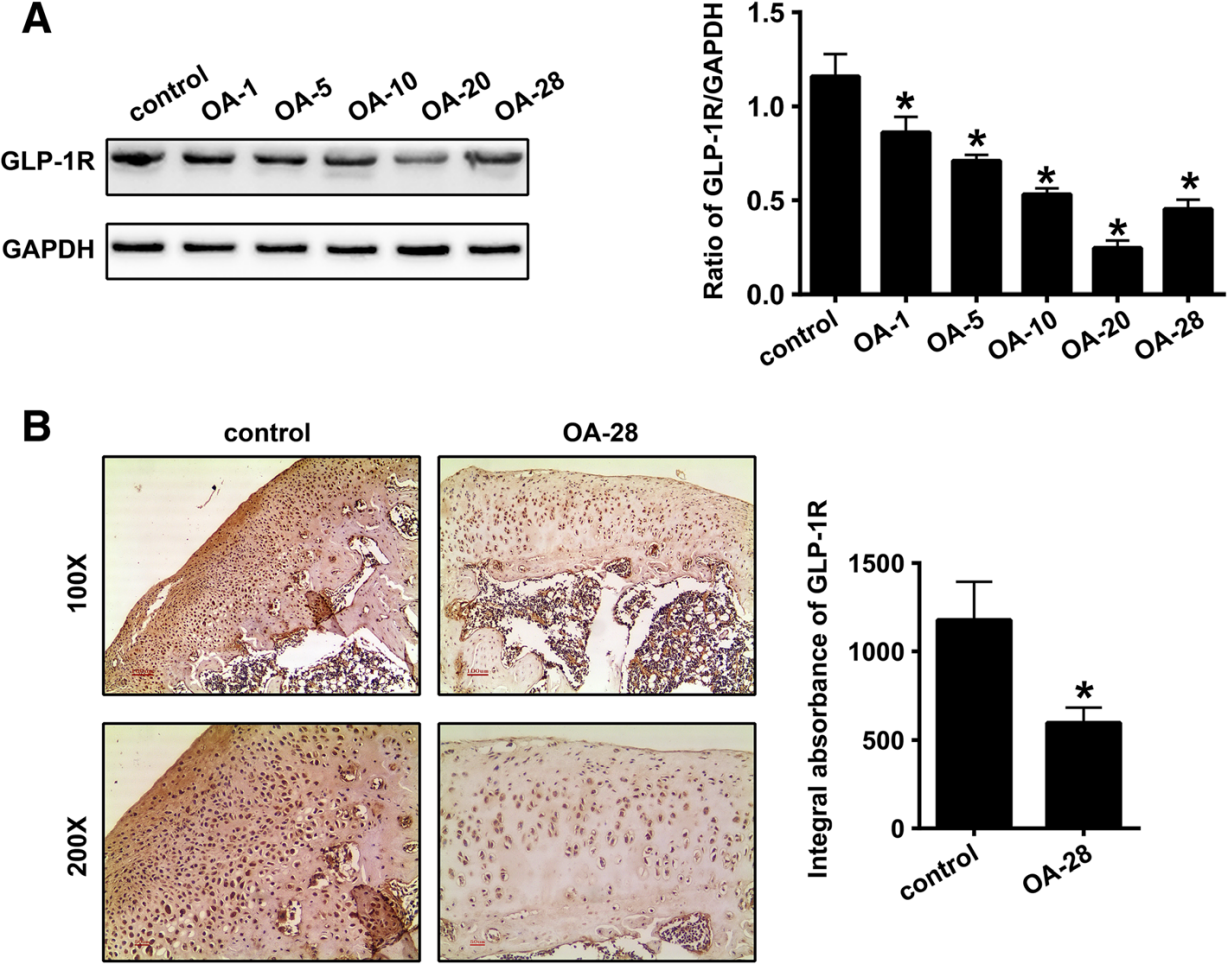
Decreased expression of GLP-1R in the cartilage tissues of a knee OA rat model. **a** Representative Western blots and quantification data of GLP-1R expression levels in control, OA-1, OA-5, OA-10, OA-20 and OA-28 groups (*n* = 5 per group). **b** Representative images (100 × and 200×) of immunohistochemical (IHC) staining and corresponding quantification of GLP-1R expression levels in the control and OA-28 groups (*n* = 5 per group). * indicates *P* < 0.05 compared with the control group

Western blot analysis was used to quantify the expression of GLP-1R in the knee cartilage tissue of the OA rats at various time-points after the induction of OA. As shown in Fig. 1a, compared to control rats, GLP-1R expression decreased significantly in the cartilage of the OA knees in the OA-1, OA-5, OA-10, OA-20 and OA-28 groups (*P* = 0.0015, *P* < 0.0001, *P* < 0.0001, *P* < 0.0001 and *P* < 0.0001, respectively). Notably, the OA-20 group presented the lowest levels of GLP-1R expression. Moreover, the levels of GLP-1R in the OA-28 group as demonstrated by IHC staining were found to be significantly downregulated compared with that in the control group (Fig. 1b; *P* = 0.0005).

### GLP-1R is involved in the PKA/CREB pathway of cartilage tissues in the knee OA rat model

**Fig. 2:**
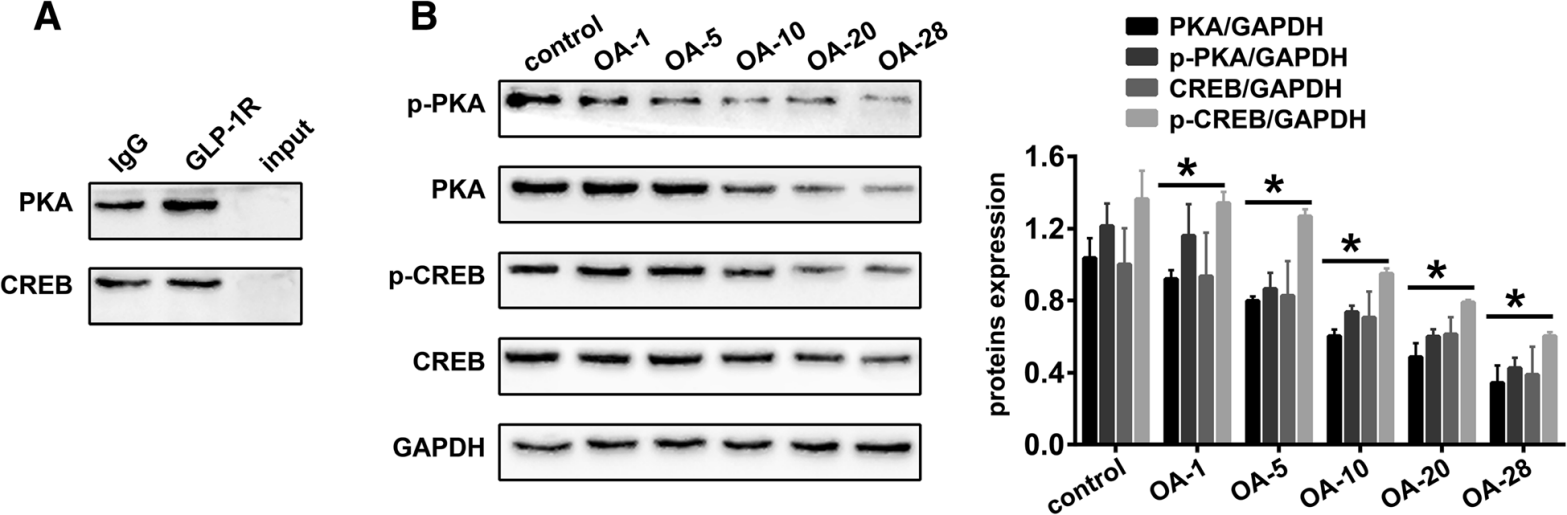
GLP-1R is associated with the PKA/CREB pathway in cartilage tissues of the knee OA rat model. **a** Representative Western blots of immunoprecipitates (n = 5 per group). **b** Representative Western blots and quantification data of PKA, p-PKA, CREB and p-CREB levels in control, OA-1, OA-5, OA-10, OA-20 and OA-28 groups (n = 5 per group). * indicates *P* < 0.05 when compared with the control group

In this study, we conducted immunoprecipitation analysis to detect protein-protein interactions. As shown in Fig. 2a, we found that GLP-1R was implicated in the PKA/CREB pathway. Moreover, significantly decreased expression of PKA (Fig. 2b, *P* = 0.0692, *P* = 0.0001, *P* < 0.0001, *P* < 0.0001 and *P* < 0.0001, respectively), p-PKA (Fig. 2b, *P* = 0.8553, *P* < 0.0001, *P* < 0.0001, *P* < 0.0001 and *P* < 0.0001, respectively), CREB (Fig. 2b, *P* = 0.9657, *P* = 0.4135, *P* = 0.0583, *P* = 0.0094 and *P* < 0.0001, respectively) and p-CREB (Fig. 2b, *P* = 0.9901, *P* = 2492, *P* < 0.0001, *P* < 0.0001 and *P* < 0.0001, respectively) was detected in the OA1, OA-5, OA-10, OA-20 and OA-28 groups of the OA rat model compared with the control group.

### Induction of knee OA is accompanied by inflammation

**Fig. 3:**
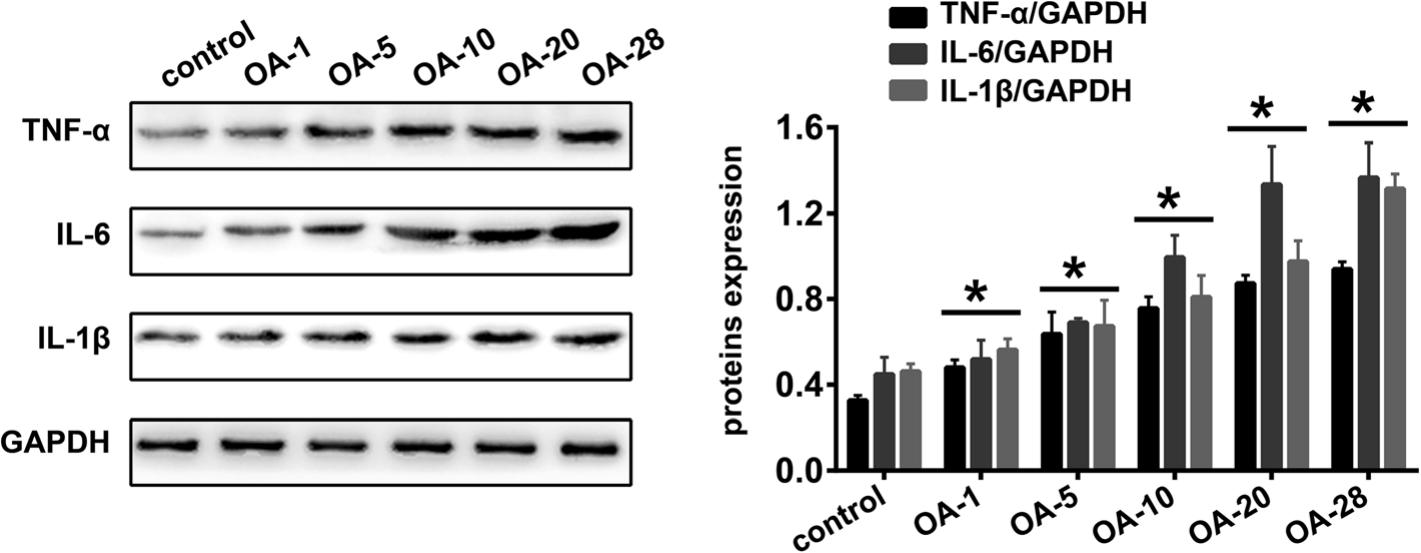
Induction of knee OA is accompanied by inflammation. Representative Western blots and quantification data for TNF-α, IL-6,and IL-1β expression levels in control, OA-1, OA-5, OA-10, OA-20 and OA-28 groups (n = 5 per group). * indicates *P* < 0.05 compared with the control group

A large number of studies have shown that inflammation plays an important role in the occurrence and development of OA. Analogously, we used Western blot analysis to detect the altered expression of the inflammation-related proteins TNF-α, IL-6 and IL-1β. As shown in Fig. 3, significantly increased expression of TNF-α (*P* = 0.0009, *P* < 0.0001, *P* < 0.0001, *P* < 0.0001 and *P* < 0.0001, respectively), IL-6 (*P* = 0.8112, *P* = 0.0144, *P* < 0.0001, *P* < 0.0001 and *P* < 0.0001, respectively) and IL-1β (*P* = 0.2403, *P* = 0.0026, *P* < 0.0001, *P* < 0.0001 and *P* < 0.0001, respectively) was detected in OA1, OA-5, OA-10, OA-20, and OA-28 groups of the OA rat model in comparison with the control group.

### Liraglutide affects body weight and upregulates GLP-1R in the cartilage tissues of the knee OA rat model

**Fig. 4:**
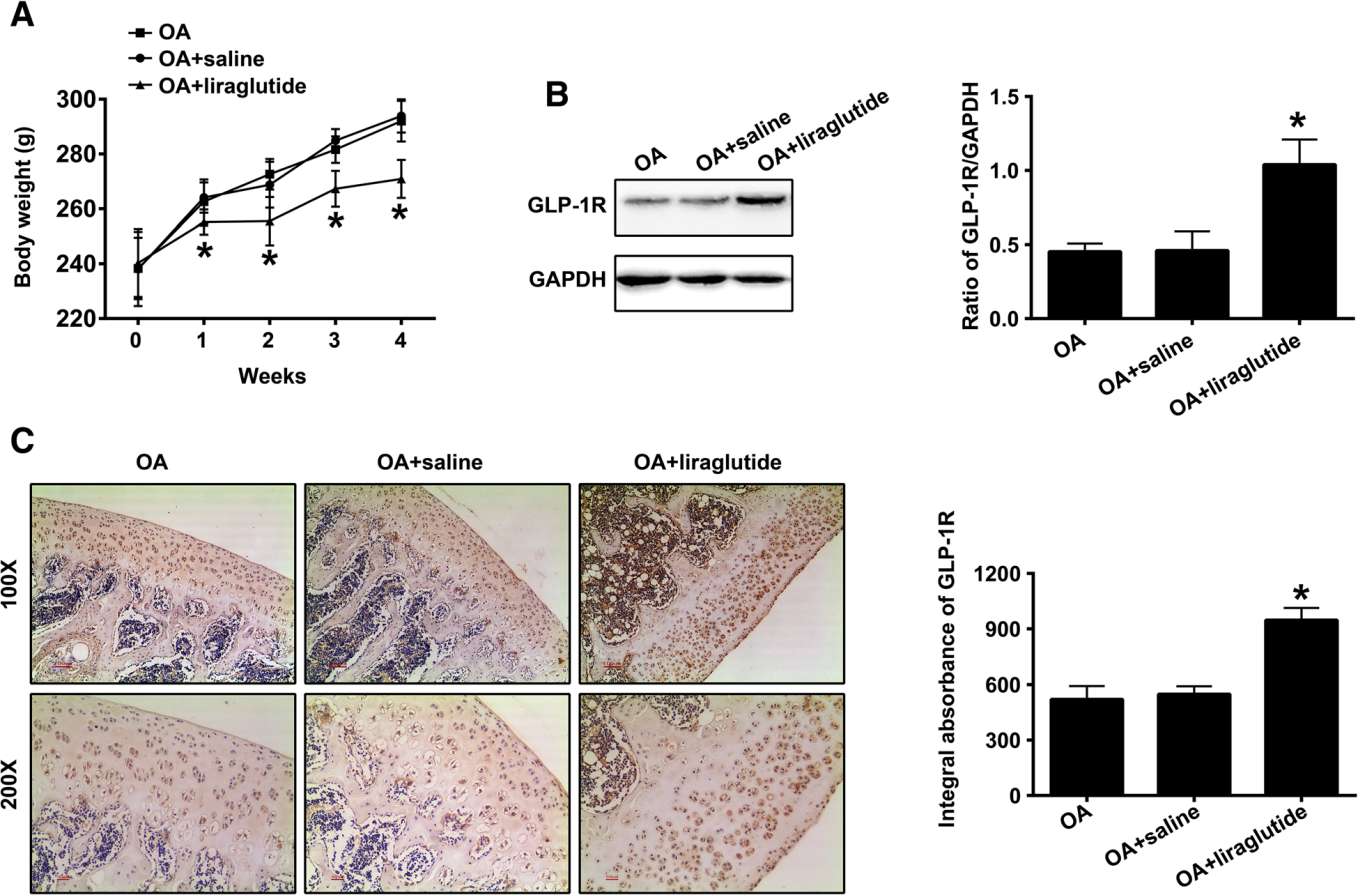
Liraglutide affects body weight and upregulates GLP-1R in the cartilage tissues of the knee OA rat mode. **a** Body weight of rats in the “OA”, “OA + saline” and “OA + liraglutide” groups at 0 day, 7th day, 14th day, 21st day and 28th day after treatment with liraglutide or saline (*n* = 5 per group). **b** Representative Western blots and quantification data of GLP-1R in the “OA”, “OA + saline” and “OA + liraglutide” groups (n = 5 per group). **c** Representative images (100 × and 200×) of IHC staining and corresponding quantification of GLP-1R levels in the “OA”, “OA + saline”, and the “OA + liraglutide” groups (n = 5 per group). * indicates *P* < 0.05 compared with the “OA + saline” group

Previous studies have indicated that liraglutide was involved in weight loss [15, 16]. Thus, we detected the body weight of rats in the “OA”, “OA + saline” and “OA + liraglutide”groups at 0 day, 7th day, 14th day, 21st day and 28th day after treatment with liraglutide or saline. As shown in Fig. 4a, compared with “OA” group, the body weight of rats in “OA + saline” had no significant change at 0 day (*P* = 0.9993), 7th day (*P* = 0.8538), 14th day (*P* = 0.5209), 21st day (*P* = 0.3575) and 28th day (*P* = 0.8092) after treatment with saline, which indicated that saline could not induce weight loss. The body weight of rats in “OA + liraglutide” had no significant change when compared with “OA + saline” group before treatment with liraglutide (*P* = 0.9310). However, compared with “OA + saline” group, the body weight of rats in “OA + liraglutide” was markedly decreased at 7th day (*P* = 0.0344), 14th day (*P* < 0.0001), 21st day (*P* < 0.0001) and 28th day (*P* < 0.0001) after treatment with liraglutide, which indicated that liraglutide could induce body loss. Western blot analysis was used to measure the expression of GLP-1R in the cartilage tissues of “OA”, “OA + saline” and “OA + liraglutide” groups after the rats induced to develop OA received 28-day treatment with liraglutide or saline. As shown in Fig. 4b, treatment with liraglutide (OA + liraglutide group) exhibited significantly increased expression of GLP-1R in the cartilage tissues of the OA knees compared with saline treatment (“OA + saline”) (*P* < 0.0001). However, there was no statistical significance in GLP-1R expression between the “OA” and the “OA + saline” groups (Fig. 4b; *P* = 0.9956). These results were also demonstrated by immunohistochemical staining and quantified accordingly (Fig. 4c; *P* < 0.0001 OA + liraglutide vs OA + saline; *P* = 0.7597 OA vs OA + saline).

### Liraglutide activates the PKA/CREB pathway in the cartilage tissues of the knee OA rat model

**Fig. 5:**
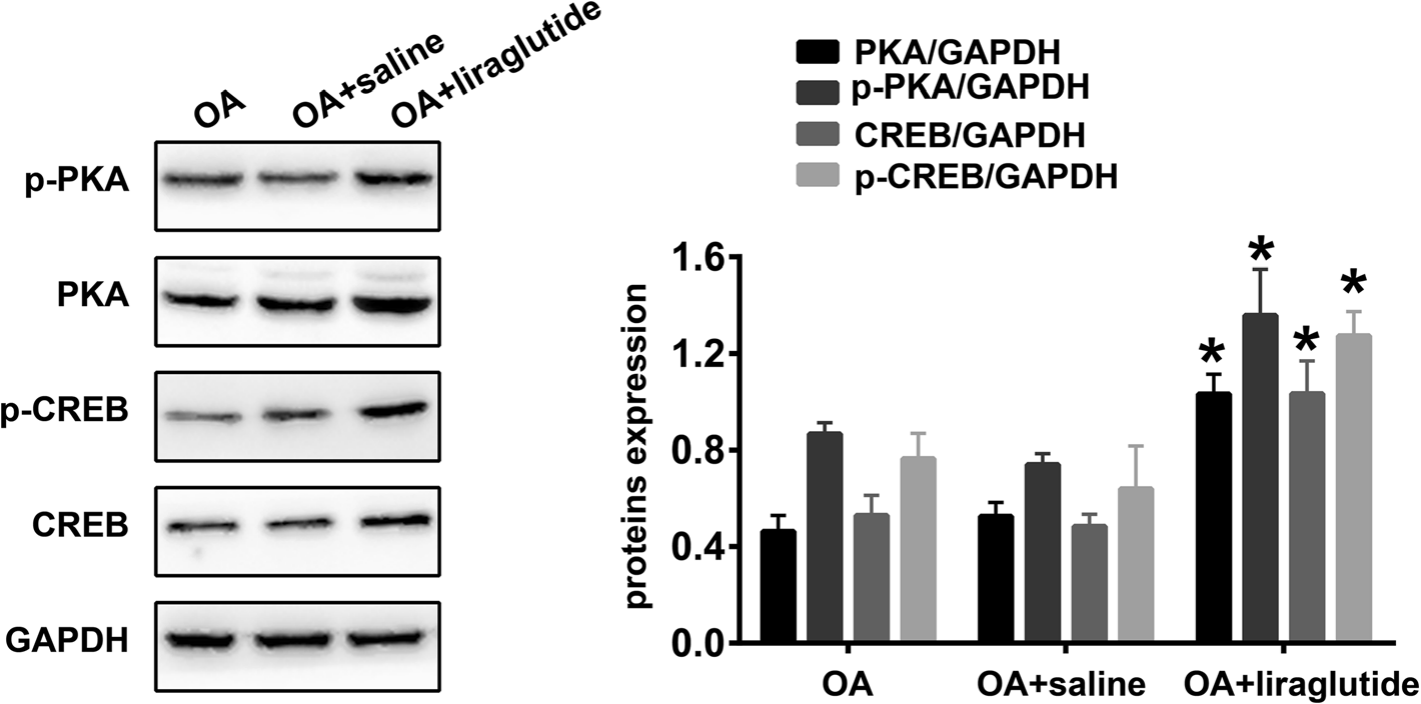
Liraglutide activates the PKA/CREB pathway in cartilage tissues of the knee OA rat model. Representative Western blots and quantification data of PKA, p-PKA, CREB and p-CREB in the “OA”, “OA + saline” and “OA + liraglutide” groups (n = 5 per group). * Indicates *P* < 0.05 compared with the “OA + saline” group

Western blot analysis was also used to detect the expression of PKA/CREB pathway components in the cartilage tissues of OA knees after treatment with liraglutide or saline. As shown in Fig. 5, treatment with liraglutide (“OA + liraglutide” group) significantly increased the expression of PKA (*P* < 0.0001), p-PKA (*P* < 0.0001), CREB (*P* < 0.0001) and p-CREB (*P* < 0.0001) in the cartilage tissues of OA knees compared with treatment using saline (“OA + saline”). However, there were no statistical differences in PKA (*P* = 0.3573), p-PKA (*P* = 0.2439), CREB (*P* = 0.7447) or p-CREB (*P* = 0.3302) expression levels between the “OA” and “OA + saline” groups.

### Liraglutide inhibits inflammation in the knee OA rat model

**Fig. 6:**
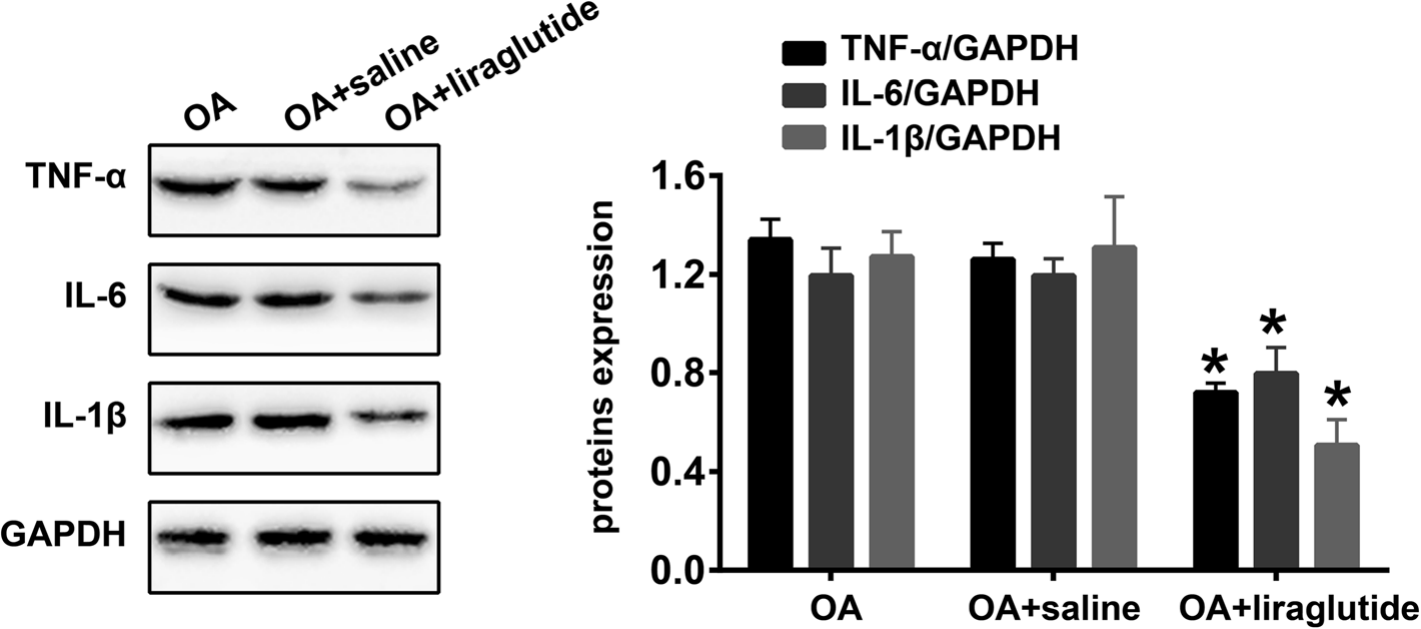
Liraglutide ameliorates inflammation in the knee OA rat model. Representative Western blots and quantification data for TNF-α, IL-6 and IL-1β in the “OA”, “OA + saline” and “OA + liraglutide” groups (n = 5 per group). * indicates *P* < 0.05 compared with the “OA + saline” group

Finally, we also quantified the expression of the inflammation-related proteins TNF-α, IL-6 and IL-1β. As shown in Fig. 6, significantly decreased expression of TNF-α (*P* < 0.0001), IL-6 (*P* < 0.0001) and IL-1β (*P* < 0.0001) was detected in the cartilage tissues of the “OA + liraglutide” group compared with the “OA + saline” group. However, there were no statistically significant differences in TNF-α (*P* = 0.1696), IL-6 (*P* = 0.9869) and IL-1β (*P* = 0.9179) expression levels between the “OA” and “OA + saline” groups (Fig. 6).

## Discussion

In the present study, we found that GLP-1R protein levels decreased significantly in a knee OA rat model induced by monoiodoacetate, which was accompanied by the downregulation of the PKA/CREB pathway and upregulation of the inflammation-related proteins TNF-α, IL-6 and IL-1β. We also found that GLP-1R interacted with the PKA/CREB pathway and that liraglutide could activate PKA/CREB signals and inhibit the expression of inflammation-related proteins. Together these data indicate that liraglutide exhibits anti-inflammatory activity through the activation of the PKA/CREB pathway in an OA rat model. In addition, we also found that liraglutide could cause weight loss during treatment, which may be closely related to its mechanism in weight regulation. However, whether this weight loss had a bad effect on OA rats remained to be further studied.

OA was once defined as a non-inflammatory arthropathy, but it is now well-recognized that there is a major inflammatory component to this disease. Previous studies indicated that inflammation plays an important role in the development of OA, which is often closely related to low-grade synovitis [17]. Moreover, cytokines produced in innate immune responses, such as TNFα, IL-6 and IL-1β are found at comparable levels in rheumatoid arthritis joint tissues, early OA and end-stage disease synovium [18]. Analogously, in our study, we also found increased levels of those cytokines in the cartilage tissues of our knee OA rat model, suggesting that chondritis might also be implicated in the occurrence and development of OA. Thus, blocking inflammatory pathways during the early stages of OA could, at least in part, delay or impede the progress of the disease, which offers a reasonable basis for the exploitation of anti-arthritis drugs. For example Ran et al., suggested that schisandrin B might be a potential therapeutic agent in the treatment of OA due to its anti-inflammatory effects via suppression of the nuclear factor-kappa B (NF-κB) and mitogen-activated protein kinase (MAPK) signal pathways [19].

Inflammation is mediated by many upstream and downstream molecules. Among them, GLP-1/GLP-1R signals have been investigated as treatment targets for inflammation-related diseases, given their anti-inflammatory functions. GLP-1 is an incretin hormone, produced by L cells to regulate glucose and energy homeostasis via GLP-1R binding [9]. GLP-1R is a G-protein coupled receptor and is widely localized in astrocytes, neurons and endothelial cells [20–22]. In the musculoskeletal system, Chen et al. found that GLP-1R was also expressed in chondrocytes and was associated with the degeneration of cartilage. Moreover, the authors also found decreased expression of GLP-1R in an OA rat model, where OA was induced by ACL resection in a transected ACL and medial menisci in the right knee joint [10]. Similarly, we also found that the expression of GLP-1R was lower in knee cartilage tissues with OA induced by the administration of monoiodoacetate compared with the control group, which suggests a potential and crucial role of GLP-1R in OA development. Although, the molecular mechanisms responsible for the anti-inflammatory effects of GLP-1/GLP-1R signals are still under investigation, the PKA/CREB pathway axis and associated active components may be major signals responsible for the anti-inflammatory effects of GLP-1/GLP-1R [23]. In cartilage tissues of the OA knee, upregulation of inflammation-related proteins is accompanied by the downregulation of GLP-1R and PKA/CREB pathways. In addition, we used protein immunoprecipitation to verify the interaction between GLP-1R and the PKA/CREB pathway. Thus, we hypothesized that GLP-1R might participate in the regulation of PKA/CREB signals, which are likely to be involved in inflammatory processes. To test this hypothesis, liraglutide, an agonist of GLP-1/GLP-1R, was used to upregulate GLP-1R signals in our OA rat model. Surprisingly, we found that liraglutide could activate the PKA/CREB pathway and inhibit inflammation. After GLP-1 binding to GLP-1R, this complex could upregulate cAMP thereby activating the PKA/CREB pathway [24]. CREB binds to CREB-binding protein (CBP), a member of the family of histone acetyltransferases (HATs), and catalyzes histone acetylation, thereby switching chromatin from a closed to an open state [12]. This change promotes the binding of RNA polymerase II and basal transcription factors to the open DNA to initiate transcription, involving anti-inflammatory cytokines, such as IL-10 and dual specificity phosphatase 1 (DUSP1) [25]. One key action of IL-10 is to transform macrophages of an M1 “inflammatory” phenotype that produce high levels of pro-inflammatory cytokines and low levels of anti-inflammatory cytokines, to an M2b “regulatory” phenotype that produce low levels of pro-inflammatory cytokines and high levels of anti-inflammatory molecules [1, 26]. DUSP1 restricts activation of the MyD88 signaling pathway by dephosphorylation and inactivation of p38 MAPKs and c-Jun N-terminal kinases (JNKs), which can be induced by glucocorticoids [26, 27]. These data suggest that the activation of CREB signals plays an important role in the anti-inflammatory response, with GLP-1R being implicated in this process.

## Conclusions

This study provides mechanistic evidence to demonstrate the anti-inflammatory effects of GLP-1R activation induced by liraglutide in a monoiodoacetate-induced knee OA rat model through the activation of the PKA/CREB pathway. This novel finding of the anti-inflammatory effects of GLP-1R in the OA knee provides promising and novel targets for the development of efficacious treatments for OA.

## Data Availability

The datasets used and/or analyzed during the current study are available from the corresponding author by reasonable request.

## Abbreviations

AC: Adenylyl cyclase
BCA: Bicinchoninic acid
cAMP: Cyclic adenosine monophosphate
CREB: Cyclic adenosine monophosphate response element-binding protein
ER: Endoplasmic reticulum
GAPDH: Glyceraldehyde 3-phosphate dehydrogenase
GLP-1: Glucagon-like peptide-1
GLP-1R: Glucagon-like peptide-1 receptor
HPRT1: Hypoxanthine phosphoribosyl transferase 1
HRP: Horseradish peroxidase
IL-1β: Interleukin-1 beta
OA: Osteoarthritis
PI3K: Phosphoinositide 3-kinase
PKA: Protein kinase A
RIPA: Radio immunoprecipitation assay
ROS: Reactive oxygen species
TNF-α: Tissue Necrosis Factor-alpha
